# The importance of the neutrophil‐to‐lymphocyte ratio in the prognosis of glioma and its subtypes

**DOI:** 10.1111/cns.13270

**Published:** 2019-11-15

**Authors:** Paulo Linhares, Axel Ferreira, Rui Vaz

**Affiliations:** ^1^ Serviço de Neurocirurgia do Centro Hospitalar São João Porto Portugal; ^2^ Faculdade de Medicina da Universidade do Porto Porto Portugal; ^3^ Unidade de Neurociências do Hospital CUF Porto Porto Portugal; ^4^ Serviço de Neurologia do Hospital Pedro Hispano Matosinhos Portugal

Dear editor

We read with great interest the original article by Wang Z. et al that found a correlation between neutrophil‐to‐lymphocyte ratio (NLR) and glioma prognosis. His group detected, in their patient cohort, a correlation between NLR <3.2 and better prognosis and also between NLR >3.2 and poor prognosis.^1^ The subdivision of these tumors between neutrophil type and lymphocyte type could contribute to a better stratification of patient prognosis with a simple and relatively innocuous blood test.

In fact, it is known for many years that inflammation promotes angiogenesis and proliferation of malignant cells.^2^ In that matter, knowing that NLR is a marker of inflammation, it is logical that NLR can be an independent risk factor for worse prognosis in several malignancies.^3^, ^4^ And, as it is acknowledged by the mentioned paper, similar findings have been made before in gliomas.^5^, ^6^ Nonetheless, relating NLR values with WHO grades of malignancy can be a limitative way of using this potential new tool. It is already established that different types of glioma have different prognosis, and the endeavor of the WHO classification, especially the 2016 update, is exactly that, trying to classify tumors in subtypes and grade them according to prognosis.^7^ In that respect, we think that the use of NLR should not be restrained to predict the grade of a glial tumor.

Following this train of thought, we feel that the discussion should be centered in the effect of NLR values in the prognosis of the different glioma subtypes, specifically the ones that are more common and aggressive. Much like the discovery of the relation between IDH1 mutations and better prognosis in glioblastoma (GBM),^8^ we think that the NLR can have an analogous role in certain glioma subtypes. Interestingly, recent research by Auezova et al have shown that IDH1 mutations in glioma are correlated to lower NLR values. As NLR is a marker for chronic inflammation, they infer that IDH1 mutations are related to lower levels of chronic inflammation, and therefor to a better prognosis.^9^ In retrospective, we think that the main constraint of the Wang Z. et al paper is the fact that all grades of glioma were considered and, for us, the question is not what is the behavior of NLR among the grades, but what we can achieve among the same grade.

In previous work made by our group and published in the Journal of Neuro‐oncology, we analyzed a cohort of 140 patients with GBM submitted to resection surgery between January 2005 and January 2013 in a tertiary care hospital. As adjunctive treatment, 117 patients completed the Stupp protocol. As for the remaining patients, 14 patients did no adjunctive therapy and 9 underwent only standard or hypofractionated radiotherapy, or only chemotherapy with Temozolomide or Carmustine due to poor functional status. Mean age at surgery was 62.9 ± 10.0 years, and mean age at death was 64.4 ± 9.8 years. Mean overall survival was 19.4 ± 14.3 months, and mean progression‐free survival was 9.4 ± 8.7 months. In the complete cohort, we did not find a correlation between NLR and overall survival; however, in the subgroup of 117 patients that completed Stupp protocol (radiotherapy and chemotherapy with Temozolomide), we found that a preoperative NLR >7 correlated with a shorter overall survival (Figure 1).^10^ Other groups have found similar results.^11^, ^12^, ^13^, ^14^ Most significantly Bambury et al found, in a cohort of 84 GBM patients, that preoperative NLR >4 was associated with worse median overall survival.^11^ And McNamara et al published identical conclusions in a cohort of 107 GBM patients undergoing second surgery.^12^ All the prementioned studies had their limitations, in the sense that they were all retrospective and therefore prone to bias. In that matter, we feel that prospective studies should be done to confirm this finding. However, we feel that the main goal should be guided toward studying specific subtypes, one because different types of glioma have different known prognosis, which renders an holistic sample of little utility, and two because many of the observed cases will be of GBM, which has an already known relation between NLR and prognosis, and can contaminate the sample.

**Figure 1:**
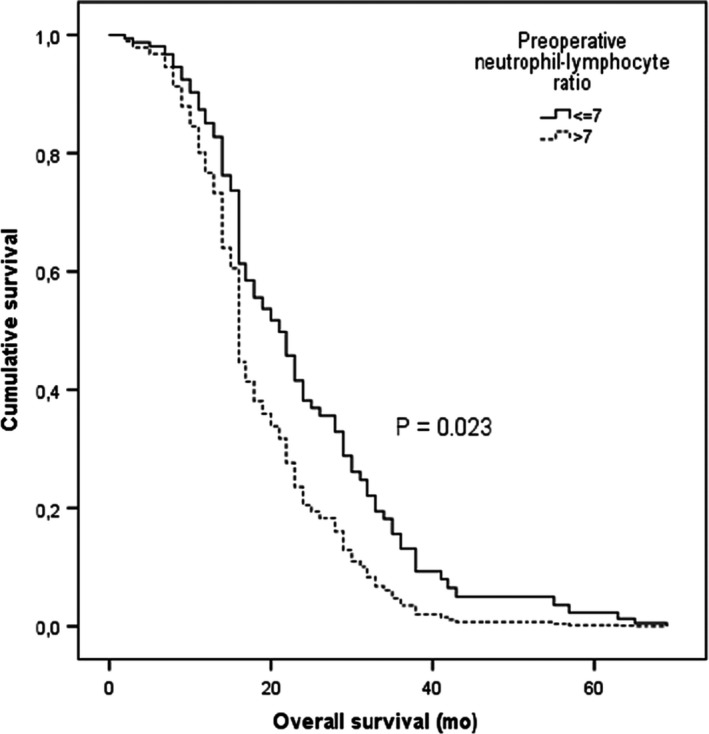
Kaplan‐Meier plot of overall survival stratified by neutrophil‐lymphocyte ratio value (cut‐off =7.0)—Stupp protocol treated patients

Interestingly, our research also found that the relation with prognosis was only significant in the patients that followed optimal therapy. This can suggest that parameters like NLR may, in the future, modulate therapy options, because it implies that a more aggressive approach can be futile in more aggressive types of GBM.

As it has been said, we think that the use of NLR shouldn’t be limited to predicting the grade of malignancy in glial tumors. In the greater picture, it can be considered as a simple easy access tool to better establish prognosis in certain glioma subtypes, as it appears to be the case in GBM, and really change, in a daily basis, our therapeutic approach.

## CONFLICT OF INTEREST

The authors have no disclosures or conflict of interest to declare.
